# MicroRNA-143 down-regulates Hexokinase 2 in colon cancer cells

**DOI:** 10.1186/1471-2407-12-232

**Published:** 2012-06-12

**Authors:** Lisa B Frankel, Jiayu Wen, Anders Krogh, Anders H Lund

**Affiliations:** 1 Biotech Research and Innovation Centre and Centre for Epigenetics, University of Copenhagen, DK-2200, Copenhagen N, Denmark; 2 The Bioinformatics Centre, Department of Biology, University of Copenhagen, DK-2200, Copenhagen N, Denmark; 3 Berlin Institute for Medical Systems Biology, Max-Delbrück-Center for Molecular Medicine, D-13125, Berlin, Germany; 4 Computational Biology Program, Memorial Sloan-Kettering Cancer Center, New York, NY, USA

**Keywords:** miR-143, Colon cancer, Hexokinase 2, Glycolysis

## Abstract

**Background:**

MicroRNAs (miRNAs) are well recognized as gene regulators and have been implicated in the regulation of development as well as human diseases. miR-143 is located at a fragile site on chromosome 5 frequently deleted in cancer, and has been reported to be down-regulated in several cancers including colon cancer.

**Methods:**

To gain insight into the role of miR-143 in colon cancer, we used a microarray-based approach in combination with seed site enrichment analysis to identify miR-143 targets.

**Results:**

As expected, transcripts down-regulated upon miR-143 overexpression had a significant enrichment of miR-143 seed sites in their 3'UTRs. Here we report the identification of Hexokinase 2 (HK2) as a direct target of miR-143. We show that re-introduction of miR-143 in the colon cancer cell line DLD-1 results in a decreased lactate secretion.

**Conclusion:**

We have identified and validated HK2 as a miR-143 target. Furthermore, our results indicate that miR-143 mediated down-regulation of HK2 affects glucose metabolism in colon cancer cells. We hypothesize that loss of miR-143-mediated repression of HK2 can promote glucose metabolism in cancer cells, contributing to the shift towards aerobic glycolysis observed in many tumors.

## Background

MicroRNAs (miRNAs) represent an abundant group of small non-coding RNAs that repress translation and promote degradation of their mRNA targets through binding to partially complementary regions in the 3'UTR [1-3]. The target recognition is mediated by the RNA-induced silencing complex (RISC) with AGO2 as a key component. AGO2 presents the miRNA to its targets in such a way that the nucleotides at position 2–8 of the mature miRNA, also known as the seed region, are able to base pair with complementary regions in the 3'UTR [1].

Past years research on miRNAs has revealed a role of miRNAs in the regulation of numerous cellular functions including development and differentiation, cell cycle regulation, metabolism and apoptosis [4,5]. A large number of miRNAs are encoded by genes located in regions frequently exposed to changes in cancer cells [6] and alterations of miRNA expression levels have been associated with various types of cancer [7]. In addition, miRNA signatures of cancer have also in some cases been shown to correlate with the prognosis and progression of cancer [8]. By down-regulation of protein-encoding genes either promoting or inhibiting cell proliferation, several miRNAs have been shown to function as tumorsuppressors and oncogenes [5,8-11].

miR-143 is located at a fragile site often deleted in cancers [12] and has accordingly been found down-regulated in a number of cancers [13-22]. Furthermore, miR-143 overexpression has been demonstrated to have a growth inhibitory effect in several cell lines, indicating that loss of miR-143 expression could contribute to the development of cancer [13-15,18,22,23].

During development miR-143 expression has been reported to be induced during differentiation of adipocytes and vascular smooth muscle cells [24-26]. In vascular smooth muscle cells miR-143 inhibition was found to increase the proliferative potential 2-fold, but by itself miR-143 overexpression was not able to induce vascular smooth muscle differentiation [26]. This suggests that miR-143 may normally function to restrict the proliferative potential of differentiated cells, explaining why down-regulation or loss of miR-143 can contribute to the formation and/or growth of cancer.

To investigate the function of miR-143 as a putative tumorsuppressor, we sought to understand the mechanistic basis for the involvement of miR-143 in cancer by the identification of miR-143 targets. We chose to focus our study on colon cancer, since miR-143 has been frequently reported as down-regulated in colon cancers [14,15,17,18,20]. In order to identify miR-143 targets we used a microarray-based approach. Potential miR-143 targets were identified as genes containing miR-143 seed sites in the 3'UTRs that were down-regulated upon miR-143 overexpression. Here, we report that miR-143 targets and down-regulates the glycolytic enzyme hexokinase 2 (HK2) in colon cancer cell lines. Furthermore we show, that re-introduction of miR-143 leads to a decrease in lactate secretion, indicating that miR-143-mediated downregulation of HK2 impairs the rate of glycolysis.

## Methods

### Cell cultures and cell proliferation assays

Cells were cultured as previously described [27]. Overexpression of miR-143 was achieved by transient transfection with a miR-143 duplex that mimics the mature miR-143 duplex (PM10883; Ambion, Austin, TX, USA). Transfection with a scrambled negative control siRNA (1027281, Qiagen, GermantownMD, USA) was used as control. All transfections were carried out using Lipofectamine™ 2000 Transfection Reagent (11668-019, Invitrogen, Burlington, ON, Canada) according to the manufactures protocol using a final concentration of 50nM of oligonucleotides. Crystal violet assays were performed as previously described [27].

### Quantitative RT-PCR

Total RNA was isolated with TRIZOL (15596-026, Invitrogen) and treated with DNaseI (DNase-free kit™, AM1906, Ambion). Primer sequences used for quantitative PCR (Q-PCR) are listed in Additional file 1: Table S1. Hypoxanthine phosphoribosyltransfease (*HPRT*) or beta-actin (*ATCB*) served as housekeeping normalization controls. Mature miR-143 levels were quantified using TaqMan® MicroRNA Assay (4373134, Applied Biosystems, Austin, TX, USA) and normalized to the U6 small nuclear B non-coding RNA (4373381, Applied Biosystems).

### Microarray profiles

DLD-1 cells were transfected with miR-143 duplex or mock transfected in four biological replicates. Total RNA was isolated with TRIZOL 24 h after transfection. Affymetrix microarray analysis (HG-U133 Plus 2.0 human) was performed at the Microarray Center, Rigshospitalet, Copenhagen University Hospital as previously described [28]. Data processing and word analysis are described in a separate section below.

### Vector construction and reporter assays

The miR-143 luciferase reporter vector was cloned by inserting a site with perfect complementarity to miR-143 into HindIII/SpeI sites of pMIR-REPORT (AM5795, Applied Biosystems). Antisense and sense oligonucleotide sequences (with restriction overhangs indicated in lower case) are as follows:

miR-143 AS: 5'-ctagtGAGCTACAGTGCTTCATCTCAGCTCAGCA-3',

miR-143 S: 5'-agcttGCTGAGCTGAGATGAAGCACTGTAGCTCA-3',

3' UTR fragment of HK2 was PCR amplified from DLD-1 genomic DNA and cloned into the pGL3+ vector described previously [28]. The primer sequences used for PCR amplification were as follows (restriction sites indicated in lower case):

HK2 3'UTR BglII FW: 5'-GGGagatctGGAGGGATGAGAGTGGCTTA-3'

HK2 3'UTR XhoI RV: 5'-GGGctcgagAATGACAACATCTTCACTAGACTGAG-3'

The miR-143 8mer seed site, TCATCTCA, in the 3'UTR of HK2 was converted into TCATGACA using the QuikChange site-directed mutagenesis kit (Stratagene, La Jolla, CA, USA). Mutagenesis primers used were as follows:

HK2 mut FW: 5'-GTGTGATGAATAGCGAATCATGACAAATCCTTGAGCACTCAGTC-3'

HK2 mut RV: 3'-GACTGAGTGCTCAAGGATTTGTCATGATTCGCTATTCATCACAC-5'

Luciferase assays were performed as previously described [27]. Briefly, cells were co-transfected with indicated luciferase reporters and a *Renilla* normalization control, pRL-TK (E2241, Promega, Madison, WI, USA) vector alone or with miR-143 duplex or a scrambled negative control. Firefly and *Renilla* luminescence was measured 24 h after transfection using the Dual-Glo luciferase kit (E2940, Promega).

### HK2 siRNA knockdown experiments

Knockdown experiments were performed by transient transfection of HK2 siRNA using lipofectamine as described above. Cells were double transfected with 50nM siRNA for 6 h on two subsequent days and the cell lysates were harvested 48 h after the first transfection for protein and RNA extraction. The sequence of the HK2 siRNA is as previously published [29]: HK2 sense 5'-GGAUAAGCUACAAAUCAAA[dT][dT]-3',

### Antibodies and western blot analysis

For western blotting DLD-1 or HCT116 cells were double transfected for 6 h on two subsequent days. Cells were harvested 48 h after the first transfection, washed twice in PBS, and lysed in RIPA buffer (150 mM NaCl, 1% NP40, 0.5% sodium deoxycholate, 0.1% SDS, 50 mM Tris-HCl at pH 8, 2 mM EDTA) containing protease inhibitor cocktail (04693124001, Roche, Basel, Switzerland)) and phosphatase inhibitors (1 mM NaVO_3_, 10 mM NaF and 1 mM β-glycerolphosphat). 30 μg protein/lane was separated on polyacrylamide gels, transferred to a nitrocellulose membrane and incubated with antibodies against HK2 (1:1000, 2106, Cell Signaling Technology, Danvers, MA, USA) or antibodies against Tubulin (ab11304, Abcam, Cambridge, MA, USA) serving as a loading control. Band intensities were quantified using TotalLab image analysis software.

### Lactate assay

DLD-1 cells were double transfected for 6 h on two subsequent days. To measure the secretion of lactate, media samples were removed in 6 h intervals following the addition of fresh media after the second transfection and stored at −80°C until measurement. Lactate was measured using the Lactate Acid Assay Kit (K607-100, BioVison, Mountain View, CA, USA).

### Analysis of microarray profiles, seed site enrichment and word analysis

The microarray data was processed as previously described [27]. Non-specific filtering was used to remove genes with low variance between arrays using a cutoff of 0.25. This left 1241 genes that were used for the following analysis. Differentially expressed genes were found using limma [30]. Genes with a FC above 1.1 or below −1.1 were used to define the up and down set, respectively. The no-change set was selected from genes with a logFC centered on 0. The microarray data has been deposited in the GEO database accession number GSE33420.

Seed site enrichment was calculated by scanning the 3'UTR sequences in the up, down and no-change sets for the presence of 6mer, 7mer, 7mer-A1 and 8mer seed sites.

We used Gene Set Enrichment Analysis (GSEA) to detect significantly enriched biological functions/pathways from the KEGG pathway [31], Biocarta pathway (http://www.biocarta.com) and MSigDB [32] databases for the down-regulated gene sets after over-expressing miR-143. GSEA can detect an overall change in a gene set for up- and down-regulated genes even though individual genes in the set may not be significantly differentially expressed. The analysis was based on expression fold changes between miR-143 and mock transfection of all genes on the array without any cutoffs, and p-value less than 0.01 was used for statistical significance. The package “gage” [33] in Bioconductor was used for the analysis.

For the word analysis we used a non-parametric statistical framework for scoring and ranking oligonucleotide words based on their overrepresentation in a ranked list of sequences as previously described [34].

### TCGA colorectal adenocarcinoma expression correlation

Data was obtained from the public open-access HTTP directory at the TCGA website (http://tcga-data.nci.nih.gov/) for the colon adenocarcinoma (COAD) and rectum adenocarcinoma (READ) projects. Level 3 normalized Agilent microarray mRNA expression data and miRNA expression sequencing data summarized for each mature miRNA was obtained for 184 colon and rectum adenocarcinoma samples.

## Results

**Figure 1:**
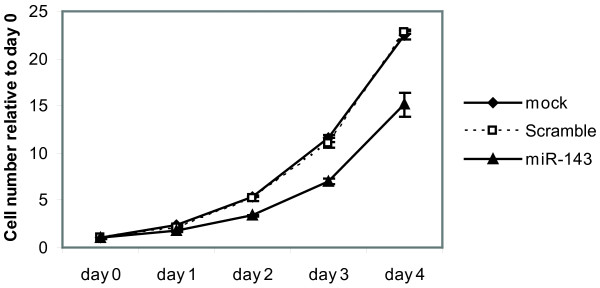
**miR-143 overexpression reduces the proliferative potential of DLD-1 cells.** DLD-1 cells transfected with miR-143 duplex exhibit a reduced cell proliferation as measured by crystal violet growth assay. Data are shown as the mean ± S.D. of four replicates. *, p < 0.005 using a two-tailed t-test, ***, p < 0.001 using a two-tailed t-test.

To investigate the expression level of miR-143 in established cell lines, we profiled miR-143 expression level using Q-PCR in a number of selected cancer cell lines as well as non-tumourigenic cell lines (Additional file 2: Figure S1). As expected, the expression levels of miR-143 were extremely low or undetectable in all tested cancer cell lines. The highest expression levels were observed in the non-tumorigenic fibroblast cell lines BJ and Tig3. We chose to focus our further studies on the human colon cancer cell line DLD-1 since miR-143 expression was virtually absent from this cell line and thus mimics the situation reported in colon cancer tumors. To confirm previous findings that miR-143 inhibits cell growth we investigated the cell proliferation upon transient transfections with a miR-143 duplex. The effect of miR-143 duplex transfection in DLD-1 cells was confirmed by co-transfection of a luciferase reporter containing a perfect complementary site to the mature miR-143 (Additional file 2: Figure S2). As demonstrated by cell growth assays, overexpression of miR-143 resulted in a decreased cell proliferation (Figure 1).

We next sought to identify functionally relevant targets that could explain the underlying role of miR-143 in cancer. To achieve this, DLD-1 cells were transfected with miR-143 duplex or mock transfected. Total RNA was harvested 24 h post-transfection and analyzed on Affymetrix HG-U133 Plus 2.0 human arrays.

To determine whether genes down-regulated by miR-143 were related to specific cellular functions, we performed a search for enriched functional annotations as derived from KEGG and BioCarta pathway databases. In KEGG pathways, down-regulated gene sets were enriched in cell cycle (p-value = 3·10^−9^), glutamate metabolism (p-value = 3·10^−4^), N-glycan biosynthesis (p-value = 2·10^−3^) and glycolysis/gluconeogenesis (p-value = 0.01) (Table 1). In BioCarta pathways, down-regulated gene set were enriched in the mTOR signalling pathway (p-value = 7·10^−5^) and the G1/S check point pathway (p-value = 5·10^−3^) (Table 2).

**Table 1:** Enriched KEGG pathways among miR-143 down-regulated gene sets

**KEGG Pathway**	**P-value**
Cell cycle	3.12^.^10^-9^
Ubiquitin mediated proteolysis	8.99^.^10^-7^
Ribosome	1.89^.^10^-6^
Aminoacyl-tRNA biosynthesis	3^.^10^-6^
Parkinson's disease	4^.^10^-6^
Pyrimidine metabolism	1^.^10^-5^
Adherens junction	2^.^10^-5^
DNA replication	2^.^10^-5^
Proteasome	3^.^10^-5^
Glutamate metabolism	3^.^10^-4^
Thyroid cancer	8^.^10^-4^
Pathogenic Escherichia coli infection - EHEC	2^.^10^-9^
N-Glycan biosynthesis	0002
Base excision repair	0.002
Vibrio cholerae infection	0.003
p53 signaling pathway	0.003
Huntington's disease	0.004
Lysine degradation	0.004
Regulation of actin cytoskeleton	0.007
Wnt signaling pathway	0.008
Biosynthesis of unsaturated fatty acids	0.008
Glycolysis / Gluconeogenesis	0.01

**Table 2:** Enriched BioCarta pathways among miR-143 down-regulated gene sets

**BioCarta Pathway**	**P-value**
HIV-I Nef: negative effector of Fas and TNF	2.42^.^10^-6^
mTOR Signaling Pathway	7.43^.^10^-5^
Ras-Independent pathway in NK cell-mediated cytotoxicity	3.27^.^10^-4^
The IGF-1 Receptor and Longevity	1.00^.^10^-3^
Inhibition of Cellular Proliferation by Gleevec	2.70^.^10^-3^
Role of ERBB2 in Signal Transduction and Oncology	2.85^.^10^-3^
Cell Cycle: G1/S Check Point	5.09^.^10^-3^

In addition we also performed a search for enriched transcription factor and miR-143 binding motifs among miR-143 down-regulated genes. The second most significantly enriched motif in the down-regulated gene set was the miR-143 seed site (p-value = 7·10^−10^), while the most significantly enriched motif was binding site of the transcription factor E2F (Additional file 3: Table S2).

**Figure 2:**
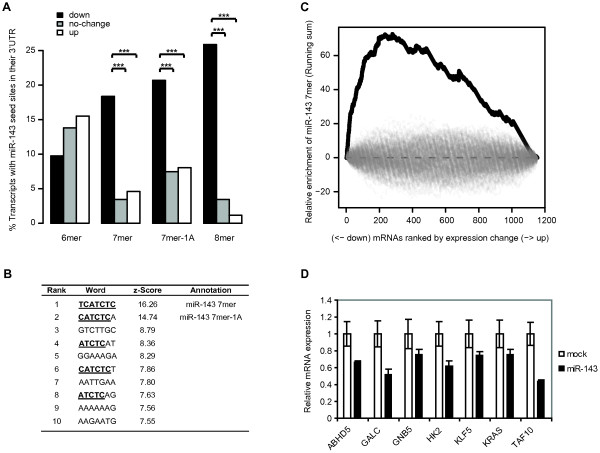
**Microarray based identification of miR-143 targets.** A, The percentages of genes in the up, down and no-change sets with seeds sites in their 3′UTRs. Seed sites were mutually exclusive. Mean log fold-changes were 0.193, −0.004 and −0.249 for the up, down and no-change sets, respectively. The p-values are calculated testing the null hypothesis that the percentages of genes with seed sites are the same for the down-regulated and the up-regulated genes (down vs. up) or the down-regulated genes compared to the no-change genes (down vs. no-change). P-values for 7mer seed site enrichment were 1.2·10 ^−4^ (down vs. up) and 3.5·10 ^−6^ (down vs. no-change). P-values for 7mer-1A seed site enrichment were 1.4·10 ^−3^ (down vs. up) and 1.8·10 ^−4^ (down vs. no-change). P-values for 8mer seed site enrichment were 2.7·10 ^−11^ (down vs. up) and 2.2·10 ^−16^ (down vs. no-change). B, Enriched 7mer words in the 3′UTRs of down-regulated transcripts. Z-scores were calculated as previously described [34]. C, An example of the unbiased word analysis (based on the 1241 genes left after non-specific filtering) showing the running sum of the overrepresentation score for the miR-143 7mer seed site TCATCTC in the list of 3′UTR sequences ranked according to their fold-change (black line) compared to permutations of the ranked gene list (grey lines). D, Quantitative RT-PCR validation of the microarray data. DLD-1 cells were transfected with miR-143 duplex or mock transfected and total RNA harvested 24 h post-transfection. The 3′UTRs of *ABHD5* and *TAF10* do not contain any miR-143 seed matches, but both genes contain a 8mer seed match in their coding region. All other miR-143 responsive genes contain at least one 7mer seed site in their 3′UTR. The expression level of each transcript data was normalized to *HPRT* and is shown relative to the level in mock transfected cells. Data are shown as the mean ± S.D. of three replicates.

Seed site enrichment analysis of seed sites present in the 3'UTRs of transcripts showed a very significant enrichment of miR-143 seed sites among the down-regulated transcripts (Figure 2A). In this analysis we grouped the 3'UTRs into down-regulated (FC < −1.1), no-change (genes with FC centred on 0) and up-regulated (FC > 1.1) (Figure 2A). The p-values for the enrichment of miR-143 seed sites (including 7mer, 7mer-1A and 8mer sites) were 3.4·10^−19^ when considering the down-regulated transcripts vs. up-regulated transcripts and 5.8·10^−28^ when considering down-regulated transcript vs. no change transcripts. This was also the case when the seed site enrichment was evaluated by two alternative methods of calculating the seed site enrichment, either as seed site occurrences after correcting the up, down and no-change sets to the same size or as seed site occurrences calculated per kb (Additional file 2: Figure S3A and S3B).

In addition to seed site enrichment analysis, we also performed an unbiased word analysis of words present in 3’UTRs of transcripts ranked according to their FC. The 7mer and the 7mer-1A seed sites of miR-143 were identified as the most significantly enriched 7mer words in the 3'UTRs of transcripts down-regulated after miR-143 overexpression (Figure 2B). Sequence variations of the miR-143 seed site were also among the highest scoring words. Similarly, a 6mer word analysis indentified the miR-143 6mer seed site as the most enriched 6mer word in 3'UTRs of down-regulated transcripts (Additional file 2: Figure S4). The overrepresentation of miR-143 seed sites in 3'UTRs of down-regulated transcripts can be visualized by plotting the running sum of the overrepresentation scores of the seed sites in transcripts ranked according to their logFC. As seen in Figure 2C the overrepresentation scores of the miR-143 7mer seed site are highest among 3'UTRs of down-regulated transcripts (black line). This was not the case, when the same analysis was performed for 100 permutations of the ranked transcript list (grey lines).

As miR-143 possesses a tumor-suppressor function, we would expect a down-regulation of oncogenes and genes promoting cell proliferation upon miR-143 overexpression. Putative miR-143 targets, defined as genes down-regulated upon miR-143 overexpression with a FC < −1.1 and containing either a least one 7mer, 7mer-1A or 8mer seed site in their 3'UTR are listed in Additional file 4: Table S3. Among the down-regulated genes containing miR-143 seed sites in their 3'UTRs we found a number of genes that have previously been implicated in tumorigenesis. This include the Steroid 5-alpha-reductase SRD5A1, the CCR4-NOT component RQCD1 and the Rab11 effector protein RAB11FIP1 which have all been reported as up-regulated in breast cancers [35-37]. Other miR-143 responsive genes with a miR-143 seed site in their 3'UTR were *SEMA5A*, *SLC35B2* and *KLF5* which have all been shown to be up-regulated in cancers and to promote cell proliferation [38-41]. Among the putative miR-143 targets we also found the deubiquitinating enzyme USP22, which have been reported to be associated with a poor prognosis of colorectal cancer [42] and invasive breast cancer [43]. In addition we also observed a reduced expression of the glycolytic enzyme *hexokinase 2* (*HK2*) upon miR-143 overexpression. HK2 catalyzes the first step of glycolysis by phosphorylation of glucose into glucose-6-phosphate. HK2 is often found upregulated in cancer and facilitates a high rate of glucose metabolism necessary for tumor growth [44].

Among genes motioned above, three genes have also been predicted by a target prediction model built on 12 transfection datasets with good prediction posterior probabilities and low FDR (<25%) [45]. This includes HK2 (posterior probability = 0.93; adjusted p-value = 0.17), RAB11FIP1 (posterior probability = 0.92; adjusted p-value = 0.17) and SEMA5A (posterior probability = 0.9; adjusted p-value = 0.22). This adds supportive evidence that these genes are direct targets of miR-143 beyond a simple seed match search.

As a validation of the microarray data we selected 7 transcripts identified as down-regulated by miR-143 in the microarray analysis for Q-PCR validation. All 7 transcripts including *HK2* were found to be down-regulated, confirming the microarray data (Figure 2D). In accordance with previous reports we also find *KRAS* downregulated upon miR-143 overexpression (Figure 2D) [15]. *KRAS* was also found down-regulated in the microarray analysis but because it had a borderline logFC of −0.14, it is not included in our list of potential miR-143 targets as listed in Additional file 4: Table S3.

**Figure 3:**
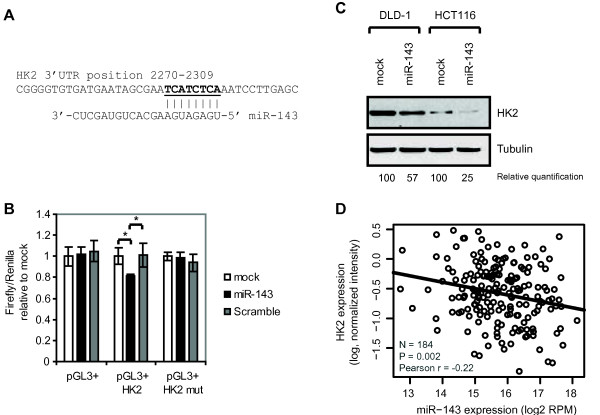
**miR-143 downregulates HK2.** A, Sequence alignment of the miR-143 seed region and HK2 3′UTR. (ENSG00000159399) B, Luciferase assay with pGL3+ constructs holding a 789 bp 3′UTR fragment of *HK2* downstream to the firefly luciferase gene. DLD-1 cells were co-transfected with firefly luciferase reporters along with a *Renilla* luciferase transfection control plasmid either alone (mock) or with miR-143 duplex and scrambled duplex as a negative control. Data are shown as the mean ± S.D. of four replicates. *, p < 0.005 using a two-tailed t-test. C, Western blot analysis of DLD-1 and HCT116 cells transfected with miR-143 duplex or mock transfected cells blotted for HK2. Tubulin was used as loading control. The bands were normalized relative to the tubulin loading control and quantified relative to the HK2 protein level in mock transfected cells. D, miR-143 and *HK2* are negatively correlated in TCGA colorectal adenocarcinoma. Linear Pearson correlation between miR-143 expression and *HK2* expression in 184 colon and rectum adenocarcinoma samples.

Due to HK2’s reported role in promoting tumor growth we wanted to investigate if the tumorsuppressor function of miR-143 can in part be accounted for due to its down-regulation of HK2. To investigate the role of miR-143 mediated regulation of HK2, we firstly wanted to determine whether HK2 is a direct target of miR-143. The 3'UTR of *HK2* contain a 8mer seed site for miR-143 (Figure 3A). To determine if miR-143 directly regulates *HK2* through binding to its 3'UTR, 3'UTR luciferase reporter constructs were cloned containing 789 base-pair UTR fragments. Overexpression of miR-143 resulted in a significant decrease of the luciferase activity (p-value < 0.002) of a construct holding the wild-type 3'UTR of *HK2* (Figure 3B). This regulation was alleviated when two nucleotides in the seed site had been mutated (Figure 3B), indicating that the miR-143 directly regulates *HK2*. Western blot analysis further confirmed that miR-143 overexpression lead to a down-regulation of HK2 protein levels in both DLD-1 and HCT116 colon cancer cells (Figure 3C). The effect of miR-143 overexpression in HCT116 cells measured by a miR-143 luciferase reporter was similar to that observed in DLD-1 cells (Additional file 2: Figure S2B). Notably the endogenous protein level of HK2 in DLD-1 cells is considerably higher than in HCT116 cells, but nevertheless miR-143 overexpression lead to a downregulation of HK2 protein levels in both cell lines.

To further strengthen the connection between miR-143 and HK2 we surveyed the expression levels of both miR-143 and *HK2* in data from The Cancer Genome Atlas (TCGA) consortium. TCGA is currently profiling the genomes of a large cohort of colon and rectum adenocarcinomas. We found a significant negative correlation between miR-143 and *HK2* (P = 0.002, r = −0.22, Pearson correlation) in 184 public TCGA colorectal adenocarcinoma tumor samples with miRNA and mRNA expression data available (Figure 3D). This observation supports that miR-143 could target and repress *HK2* expression *in-vivo* and that *HK2* expression could be upregulated in a subset of tumors due to lower levels of miR-143.

**Figure 4:**
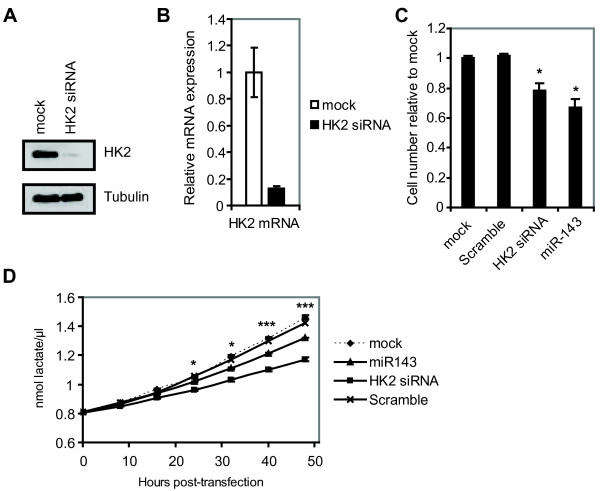
**Knockdown of HK2 as well as miR-143 overexpression results in decreased lactate secretion.** A, Verification of HK2 knockdown on protein level. Cells were transfected on two consecutive days with HK2 siRNA and protein was harvested 48 h after the first transfection. Tubulin was used as loading control. B, Verification of *HK2* knockdown on transcript level. DLD-1 cells were transfected on two consecutive days with HK2 siRNA and RNA was harvested 48 h after the first transfection. The expression level was normalized to *ATCB* and shown as the mean ± S.D. of three replicates. C, HK2 knockdown results in a reduced proliferative potential of DLD-1 cells. DLD-1 cells transfected with miR-143 duplex or HK2 siRNA exhibit a reduced cell proliferation as measured by crystal violet growth assay. Results are shown for day 4 after transfection as the mean ± S.D. of four replicates. *, p < 0.005 using a two-tailed t-test. D, Lactate secretion of DLD-1 cells is decreased upon miR-143 overexpression or HK2 knockdown compared to mock transfected cells. Cells were transfected as described in A) and B) and media samples were removed in 6 h intervals for measurement of lactate. The 0 h time points correspond to samples taken immediately after addition of fresh media following the second transfection. Data shows a representative experiment with results depicted as the mean ± S.D. of three replicates. *, p < 0.005 using a two-tailed t-test.

To investigate the effect of HK2 on cellular growth, we performed cell proliferation assay upon siRNA-mediated knockdown of HK2. HK2 knockdown was verified on both mRNA and protein level in DLD-1 cells (Figure 4A and B). We found that, in a similar manner to miR-143 overexpression, HK2 siRNA mediated knockdown also resulted in a reduced cell proliferation (Figure 4C).

Next, to determine if downregulation of HK2 mediated by miR-143 resulted in an impairment of glycolysis, lactate production was measured in mock transfected cells and cells transfected with miR-143 duplex or HK2 siRNA. Cells transfected with a HK2 siRNA showed a marked decrease in the rate of lactate secretion over a period of 48 h (Figure 4D). Importantly, a decrease in the lactate secretion was also observed upon miR-143 overexpression (Figure 4D), confirming that miR-143 downregulation of HK2 has a functional effect on the glucose metabolism. The observed decrease in lactate secretion caused by miR-143 overexpression is less pronounced than for HK2 siRNA mediated inhibition. However, this might be explained by the more efficient down-regulation of HK2 mediated by the HK2 siRNA than by overexpression of miR-143.

## Discussion

In accordance with numerous reports of miR-143 down-regulation in cancer, we observed low or undetectable expression levels of miR-143 in human cancer cell lines. This was in contrast to non-tumorigenic fibroblast cell lines, which had a relatively high expression level of miR-143. In addition, we confirmed the growth inhibitory effect of miR-143 reported by others in DLD-1 colon cancer cells [14,15,18].

Using a microarray based experimental approach we have identified a number of putative miR-143 targets that are down-regulated at the transcript level by miR-143 overexpression and contain miR-143 seed sites in their 3'UTRs. This target identification approach provides a way to indentify functionally relevant miRNA targets in colon cancer cells without any assumption concerning the conservation of miR-143 binding sites, but by means of detecting expression changes of potential miR-143 targets at the transcript level. Even though miRNAs repress the protein output of their target genes as a combined effect of mRNA destabilisation and translational repression, a recent study has reported transcript destabilization to be the main contribution to miRNA target deregulation [46]. Therefore, target identification based on detection of changes at the transcript level should in principal be able to detect the majority of miRNA targets, thus justifying our approach to identify miRNA targets. As a further confirmation of this strategy to identify miRNA targets, we observed a highly significant enrichment of miR-143 seed sites in the 3'UTRs of genes down-regulated upon miR-143 overexpression.

Among the putative miR-143 targets we found a number of genes known to promote cell proliferation, including SRD5A1, RQCD1, RAB11FIP1, SEMA5A, KLF5, USP22, SLC35B2 and HK2. Three of these genes have also been predicted as miR-143 targets by an independent miRNA target prediction algorithm [45]. Previous studies have identified ERK5 and KRAS as miR-143 targets in colon cancer [14,15]. In our study we also observed down-regulation of *KRAS* upon miR-143 transfection. However the degree of down-regulation was above the logFC of −1.1 used to define our set of putative miR-143 target. In the case of ERK5, we did not observe any change in expression in our microarray experiment. This is in agreement with a study of miR-143 in liposarcoma that also did not identify ERK5 as a miR-143 target, but did observe a down-regulation of HK2 in response to miR-143 overexpression [22].

miR-143 mediated down-regulation of one or more of the above mentioned genes in colon cancer cells could account for the growth inhibitory effect of miR-143. However, the tumor suppressive function of miR-143 is likely a result of the combined effect of miR-143-mediated down-regulation of several genes rather than a single gene. Considering miR-143 down-regulated genes in our study, including both direct and potential secondary effects, we found an enrichment of genes involved in cell cycle regulation as well as cellular metabolism. This suggests that miR-143 targets genes involved in a number of cellular pathways, including pathways controlling cell growth and metabolism which mediates downstream gene expression changes of genes in these pathways.

We chose to focus on HK2 as a potential target of miR-143 for further functional analysis, because we hypothesized that miR-143 mediated regulation of HK2 may account for the changes in glucose metabolism observed in many cancer cells. Alterations in glucose metabolism in cancer cells have been known for a long time. This was first reported by Warburg, who noted that cancer cells take up high amounts of glucose which is converted primarily into lactate and has hence been coined the Warburg effect [47]. Whereas non-proliferating cells mainly produce energy by oxidative phosphorylation, proliferative cells and cancer cells also get a significant part of their energy from aerobic glycolysis [48]. During aerobic glycolysis cancer cells convert pyruvate into lactate—a process normally inhibited by the presence of oxygen. HK2 is overexpressed in many human cancers and has been reported to be involved in maintenance of the malignant state of tumors [44]. The overexpression of HK2 in cancer is thought to provide cancer cells with a growth advantage due to increase glycolytic flux by promoting the first step of glycolysis and thus promoting/inducing the shift towards aerobic glycolysis. This type of catabolism of glucose with lactate as the end product produces significantly less ATP than oxidative phosphorylation, but even though the ATP production is reduced, this shift is thought to provide rapidly dividing cancer cells with certain advantages, such as the biosynthesis of nucleic acids as well as providing the cofactor NADPH for synthesis of phospholipids and fatty acids though the pentose phosphate pathway [48]. In addition to creating an acid environment protecting against the immune system and favouring invasion of surrounding tissue [48]. Finally, the Warburg effect also makes the cells less dependent on oxygen, which ensures survival during hypoxic and anoxic conditions.

Here, we reported the identification of *HK2* as a target of miR-143, confirming the down-regulation of HK2 upon miR-143 overexpression of transcript cells as well as protein level in both DLD-1 and HCT116 colon cancer cell lines. Interestingly the expression level of HK2 is markedly different between DLD-1 and HCT116 cells. This might be due to different mutations in the two cell lines that are giving rise to the tumorigenic phenotype or different metabolic adaptations to the need for a fast proliferation. By mutation of the miR-143 binding site in the 3'UTR of HK2 we showed that the target interaction between miR-143 and HK2 is direct. We further showed that inhibition of HK2 results in a reduction in cellular proliferation of DLD-1 colon cancer cells, an effect that resembles the effect of miR-143 overexpression. Interestingly, the decreased cell proliferation observed upon HK2 siRNA-mediated knockdown was not as strong as for miR-143 overexpression. This suggests that additional miR-143 targets besides HK2 may also be responsible for the growth inhibitory effect of miR-143. In support of miR-143’s role in glucose metabolism we showed that overexpression of miR-143 in DLD-1 cells leads to a reduced lactate secretion. However, the decrease in lactate secretion as a result of miR-143 overexpression is not as marked as the decrease observed upon HK2 siRNA mediated knockdown. This might be explained by the fact that miR-143 only mediates a relatively moderate reduction of HK2 protein level compared with the siRNA mediated knockdown of HK2.

## Conclusion

Here, we have identified a number of putative miR-143 targets in colon cancer cells. We verified HK2 as a direct target of miR-143 and show that miR-143 mediated down-regulation of HK2 results in a decreased lactate secretion. We speculate that loss of miR-143 in cancer cells might promote the metabolic shift towards aerobic glycolysis due to up-regulation of HK2.

## Competing interests

The authors declare that they have no competing interests.

## Authors’ contributions

LHG carried out experiments and analysis of the microarray data. AJ developed the word analysis tool and carried out analysis of TCGA data. LBF participated in planning and supervision of experiments. JW carried out the GSEA analysis and helped with statistical analysis. AK and AHL conceived the study and participated in its design. LHG and AHL wrote the manuscript. All authors read and approved the final manuscript.

## Pre-publication history

The pre-publication history for this paper can be accessed here:

http://www.biomedcentral.com/1471-2407/12/232/prepub

## Supplementary Material

Additional file 1 **Table S1.** Primer sequences used for quantitative RT-PCR. Click here for file

Additional file 2 **Figures S1–S4.** Format: PDF. Click here for file

Additional file 3 **Table S2.** Enriched motifs among miR-143 down-regulated genes. Click here for file

Additional file 4 **Table S3.** List of putative miR-143 targets (defined as down-regulated transcripts with at least one miR-143 7mer, 7mer-1A or 8mer seed site in their 3'UTRs). Click here for file
