# Supplementary material for: MicroRNA-143 down-regulates Hexokinase 2 in colon cancer cells
Source: BMC Cancer. 2012 Jun 12;12:232. doi: 10.1186/1471-2407-12-232 (PMC3480834; doi:10.1186/1471-2407-12-232)
Supplement: Additional file 1 — Table S1. Primer sequences used for quantitative RT-PCR. [file 1471-2407-12-232-S1.docx]

**Supplementary Table S1: Primer sequences used for quantitative RT-PCR**

| **Gene** |  | **Forward sequence** |  | **Reverse sequence** |
| --- | --- | --- | --- | --- |
| ABHD5 |  | 5’-TGTCAGCCGGCTTCGAGATAAG-3’ |  | 5’-ACCAGTTAGCCATCCTGACCTCTC-3’ |
| ATCB |  | 5’-CTCCCCGGGCTGTATTCC-3’ |  | 5’-CCTCTCTTGCTCTGGGCCTC-3’ |
| GALC |  | 5’-TCTCAACCAGAGACCCATTACGTG-’3 |  | 5’-TCCTGCAATGAACACACCTCCTG-3’ |
| GNB5 |  | 5’-TCAAACAACGAGCTCTGAGACCAG-’3 |  | 5’-CCCTCGGTTGCCATAATTTCTGC-3’ |
| HK2 |  | 5’- ACCCGGGAAAGCAACTGTTTG-3’ |  | 5’- TCACCAGGATAAGCCTCACCAG-3’ |
| HPRT |  | 5’- ACCCGGGAAAGCAACTGTTTG-3’ |  | 5’- TCACCAGGATAAGCCTCACCAG-3’ |
| KLF5 |  | 5’- ACTACTGCGATTACCCTGGTTGC-3’ |  | 5’- CCTTCCCAGGTACACTTGTATGGC-3’ |
| KRAS |  | 5’- CAGCAAAGACAAGACAGAGAGTGG-3’ |  | 5’- TGTCGGATCTCCCTCACCAATG-3’ |
| TAF10 |  | 5’-TGCCAATGATGCCCTACAGCAC-3’ |  | 5’-AGAGTGTACTTGCGGTCCTTGC-3’ |
|  |  |  |  |  |
