# Supplementary material for: MicroRNA-143 down-regulates Hexokinase 2 in colon cancer cells
Source: BMC Cancer. 2012 Jun 12;12:232. doi: 10.1186/1471-2407-12-232 (PMC3480834; doi:10.1186/1471-2407-12-232)
Supplement: Additional file 2 — Figures S1–S4. Format: PDF. [file 1471-2407-12-232-S2.pdf]

S1

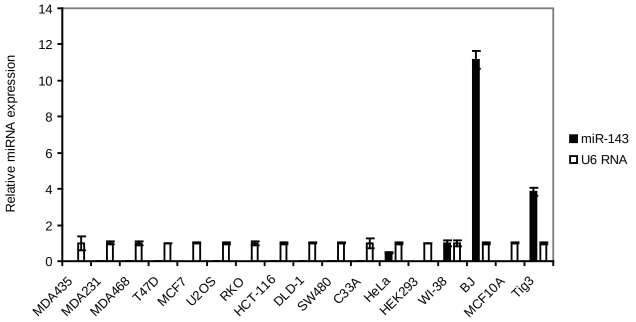

S2A

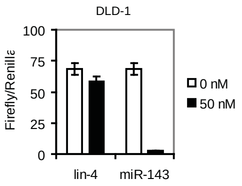

S2B

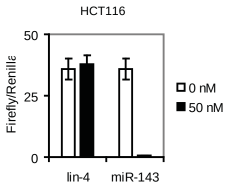

S3A

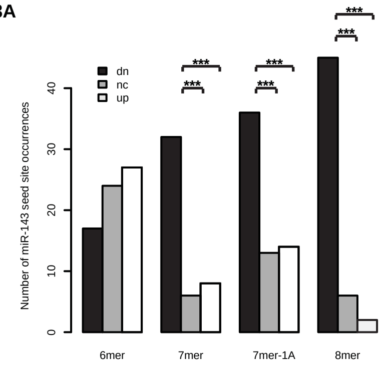

S3B

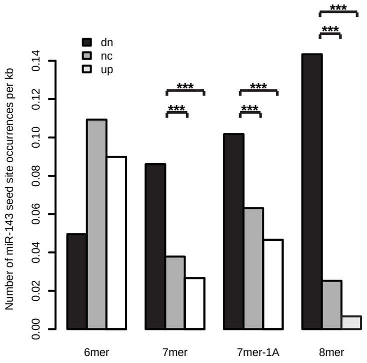

S4

Rank	Word	z-Score	Annotation
1	**CATCTC**	14.98	miR-143 6mer
2	**ATCTCA**	11.25	
3	**TCTC** AT	9.13	
4	**TCATCT**	9.11	
5	TT **ATCT**	8.75	
6	TTTCAA	8.65	
7	AACTAT	8.36	
8	ATTGAA	8.07	
9	ATTTGT	7.62	
10	CAAAAT	7.56	
