# Supplementary material for: MicroRNA-143 down-regulates Hexokinase 2 in colon cancer cells
Source: BMC Cancer. 2012 Jun 12;12:232. doi: 10.1186/1471-2407-12-232 (PMC3480834; doi:10.1186/1471-2407-12-232)
Supplement: Additional file 3 — Table S2. Enriched motifs among miR-143 down-regulated genes. [file 1471-2407-12-232-S3.docx]

**Supplementary Table S2: Enriched motifs among miR-143 down-regulated genes.**

| **Associated Transcription Factor/ miRNA** | **Standard Name** | **Motif** | **P-value** |
| --- | --- | --- | --- |
| E2F | V$E2F_Q4 | TTTSGCGS | 6.7^.^10^-12^ |
| miR-143 | TCATCTC,MIR-143 | TCATCTC | 7.1^.^10^-10^ |
| YY1 | GCCATNTTG_V$YY1_Q6 | GCCATNTTG | 9.4^.^10^-8^ |
|  | TMTCGCGANR_UNKNOWN | TMTCGCGANR | 1.1^.^10^-7^ |
| GABPA | V$GABP_B | VCCGGAAGNGCR | 2.4^.^10^-7^ |
|  | TGCGCANK_UNKNOWN | TGCGCANK | 4.2^.^10^-7^ |
|  | TAANNYSGCG_UNKNOWN | TAANNYSGCG | 1.5^.^10^-5^ |
|  | TCCCRNNRTGC_UNKNOWN | TCCCRNNRTGC | 2.9^.^10^-5^ |
|  | YGCGYRCGC_UNKNOWN | YGCGYRCGC | 3.1^.^10^-5^ |
| ELK1 | V$ELK1_02 | NNNNCCGGAARTNN | 3.3^.^10^-5^ |
| MAX | V$MAX_01 | NNANCACGTGNTNN | 1.1^.^10^-4^ |
|  | RRCCGTTA_UNKNOWN | RRCCGTTA | 1.9^.^10^-4^ |
|  |  |  |  |
