# Supplementary material for: MicroRNA-143 down-regulates Hexokinase 2 in colon cancer cells
Source: BMC Cancer. 2012 Jun 12;12:232. doi: 10.1186/1471-2407-12-232 (PMC3480834; doi:10.1186/1471-2407-12-232)
Supplement: Additional file 4 — Table S3. List of putative miR-143 targets (defined as down-regulated transcripts with at least one miR-143 7mer, 7mer-1A or 8mer seed site in their 3'UTRs). [file 1471-2407-12-232-S4.docx]

**Supplementary Table S3: List of putative miR-143 targets (defined as down-regulated transcripts with at least one miR-143 7mer, 7mer-1A or 8mer seed site in their 3’UTRs)**

| **Ensembl Gene Identifier** | **HGNC Symbol** | **logFC** | **P-value** | **FDR** | **6mer** | **7mer** | **7mer-1A** | **8mer** |
| --- | --- | --- | --- | --- | --- | --- | --- | --- |
| ENSG00000167767 | KRT80 | -0.748 | 0.002 | 0.548 | 2 | 1 | 1 | 1 |
| ENSG00000174442 | ZWILCH | -0.653 | 0.005 | 0.834 | 2 | 1 | 1 | 1 |
| ENSG00000151233 | GXYLT1 | -0.630 | 0.005 | 0.834 | 4 | 3 | 1 | 1 |
| ENSG00000198060 | 39145 | -0.625 | 0.005 | 0.834 | 3 | 1 | 1 | 0 |
| ENSG00000135052 | GOLM1 | -0.616 | 0.011 | 1.000 | 1 | 1 | 1 | 1 |
| ENSG00000101193 | C20orf11 | -0.603 | 0.008 | 0.962 | 5 | 1 | 2 | 1 |
| ENSG00000147471 | PROSC | -0.529 | 0.061 | 1.000 | 2 | 1 | 1 | 1 |
| ENSG00000183044 | ABAT | -0.526 | 0.015 | 1.000 | 3 | 1 | 1 | 0 |
| ENSG00000054983 | GALC | -0.498 | 0.025 | 1.000 | 2 | 2 | 1 | 1 |
| ENSG00000176390 | CRLF3 | -0.497 | 0.019 | 1.000 | 1 | 1 | 0 | 0 |
| ENSG00000011260 | UTP18 | -0.496 | 0.021 | 1.000 | 1 | 1 | 1 | 1 |
| ENSG00000103356 | EARS2 | -0.481 | 0.031 | 1.000 | 3 | 2 | 1 | 1 |
| ENSG00000004961 | HCCS | -0.473 | 0.026 | 1.000 | 1 | 1 | 0 | 0 |
| ENSG00000089818 | NECAP1 | -0.452 | 0.037 | 1.000 | 1 | 1 | 1 | 1 |
| ENSG00000182552 | RWDD4A | -0.446 | 0.038 | 1.000 | 1 | 1 | 0 | 0 |
| ENSG00000127838 | PNKD | -0.440 | 0.035 | 1.000 | 4 | 2 | 1 | 1 |
| ENSG00000203724 | C1orf53 | -0.432 | 0.040 | 1.000 | 1 | 1 | 0 | 0 |
| ENSG00000139146 | FAM60A | -0.431 | 0.064 | 1.000 | 1 | 1 | 0 | 0 |
| ENSG00000159596 | TMEM69 | -0.426 | 0.047 | 1.000 | 1 | 1 | 1 | 1 |
| ENSG00000168710 | AHCYL1 | -0.426 | 0.150 | 1.000 | 1 | 1 | 1 | 1 |
| ENSG00000215114 | UBXN2B | -0.417 | 0.046 | 1.000 | 3 | 2 | 1 | 1 |
| ENSG00000114573 | ATP6V1A | -0.412 | 0.089 | 1.000 | 2 | 2 | 1 | 1 |
| ENSG00000145545 | SRD5A1 | -0.411 | 0.052 | 1.000 | 1 | 1 | 1 | 1 |
| ENSG00000135387 | CAPRIN1 | -0.400 | 0.061 | 1.000 | 1 | 1 | 1 | 1 |
| ENSG00000069966 | GNB5 | -0.380 | 0.065 | 1.000 | 1 | 1 | 1 | 1 |
| ENSG00000171208 | NETO2 | -0.372 | 0.085 | 1.000 | 1 | 1 | 0 | 0 |
| ENSG00000104267 | CA2 | -0.370 | 0.080 | 1.000 | 1 | 1 | 0 | 0 |
| ENSG00000144580 | RQCD1 | -0.361 | 0.078 | 1.000 | 1 | 1 | 0 | 0 |
| ENSG00000219545 |  | -0.361 | 0.082 | 1.000 | 1 | 0 | 1 | 0 |
| ENSG00000112531 | QKI | -0.356 | 0.116 | 1.000 | 2 | 0 | 1 | 0 |
| ENSG00000135211 | TMEM60 | -0.349 | 0.087 | 1.000 | 1 | 1 | 0 | 0 |
| ENSG00000172071 | EIF2AK3 | -0.333 | 0.108 | 1.000 | 1 | 1 | 1 | 1 |
| ENSG00000154920 | EME1 | -0.314 | 0.164 | 1.000 | 1 | 1 | 1 | 1 |
| ENSG00000214049 | UCA1 | -0.312 | 0.121 | 1.000 | 1 | 1 | 0 | 0 |
| ENSG00000112902 | SEMA5A | -0.311 | 0.145 | 1.000 | 5 | 2 | 2 | 1 |
| ENSG00000109189 | USP46 | -0.305 | 0.131 | 1.000 | 1 | 1 | 1 | 1 |
| ENSG00000102554 | KLF5 | -0.300 | 0.263 | 1.000 | 1 | 1 | 1 | 1 |
| ENSG00000176142 | TMEM39A | -0.297 | 0.150 | 1.000 | 1 | 0 | 1 | 0 |
| ENSG00000205531 | NAP1L4 | -0.294 | 0.244 | 1.000 | 1 | 1 | 1 | 1 |
| ENSG00000166012 | TAF1D | -0.291 | 0.170 | 1.000 | 1 | 1 | 0 | 0 |
| ENSG00000166012 | SNORA32 | -0.291 | 0.170 | 1.000 | 1 | 1 | 0 | 0 |
| ENSG00000166012 | SNORA25 | -0.291 | 0.170 | 1.000 | 1 | 1 | 0 | 0 |
| ENSG00000169490 | TM2D2 | -0.291 | 0.170 | 1.000 | 1 | 1 | 0 | 0 |
| ENSG00000112782 | CLIC5 | -0.286 | 0.157 | 1.000 | 4 | 2 | 1 | 0 |
| ENSG00000125827 | TMX4 | -0.286 | 0.179 | 1.000 | 3 | 1 | 2 | 1 |
| ENSG00000136156 | ITM2B | -0.283 | 0.183 | 1.000 | 1 | 1 | 1 | 1 |
| ENSG00000102743 | SLC25A15 | -0.280 | 0.162 | 1.000 | 1 | 1 | 1 | 1 |
| ENSG00000093000 | NUP50 | -0.278 | 0.225 | 1.000 | 1 | 0 | 1 | 0 |
| ENSG00000129187 | DCTD | -0.277 | 0.229 | 1.000 | 1 | 0 | 1 | 0 |
| ENSG00000109685 | WHSC1 | -0.274 | 0.189 | 1.000 | 4 | 1 | 2 | 1 |
| ENSG00000196950 | SLC39A10 | -0.265 | 0.221 | 1.000 | 1 | 1 | 1 | 1 |
| ENSG00000119777 | TMEM214 | -0.262 | 0.237 | 1.000 | 3 | 1 | 2 | 0 |
| ENSG00000135913 | USP37 | -0.261 | 0.245 | 1.000 | 2 | 0 | 1 | 0 |
| ENSG00000197147 | LRRC8B | -0.260 | 0.204 | 1.000 | 2 | 1 | 1 | 1 |
| ENSG00000151552 | QDPR | -0.255 | 0.218 | 1.000 | 1 | 1 | 1 | 1 |
| ENSG00000159399 | HK2 | -0.252 | 0.252 | 1.000 | 3 | 1 | 3 | 1 |
| ENSG00000146282 | RARS2 | -0.251 | 0.232 | 1.000 | 2 | 1 | 1 | 1 |
| ENSG00000186660 | ZFP91 | -0.250 | 0.279 | 1.000 | 3 | 0 | 1 | 0 |
| ENSG00000129451 | KLK10 | -0.248 | 0.308 | 1.000 | 4 | 1 | 1 | 0 |
| ENSG00000110107 | PRPF19 | -0.247 | 0.250 | 1.000 | 1 | 1 | 1 | 1 |
| ENSG00000135245 | C7orf68 | -0.246 | 0.268 | 1.000 | 3 | 0 | 1 | 0 |
| ENSG00000142039 | CCDC97 | -0.236 | 0.262 | 1.000 | 4 | 2 | 2 | 1 |
| ENSG00000083168 | MYST3 | -0.235 | 0.295 | 1.000 | 3 | 0 | 2 | 0 |
| ENSG00000109016 | DHRS7B | -0.232 | 0.265 | 1.000 | 2 | 0 | 1 | 0 |
| ENSG00000131051 | RBM39 | -0.231 | 0.289 | 1.000 | 1 | 0 | 1 | 0 |
| ENSG00000128973 | CLN6 | -0.231 | 0.271 | 1.000 | 1 | 1 | 1 | 1 |
| ENSG00000174780 | SRP72 | -0.228 | 0.309 | 1.000 | 1 | 1 | 0 | 0 |
| ENSG00000011295 | TTC19 | -0.227 | 0.305 | 1.000 | 2 | 2 | 1 | 1 |
| ENSG00000130119 | GNL3L | -0.223 | 0.308 | 1.000 | 1 | 1 | 0 | 0 |
| ENSG00000128342 | LIF | -0.221 | 0.308 | 1.000 | 2 | 2 | 0 | 0 |
| ENSG00000160124 | CCDC58 | -0.212 | 0.312 | 1.000 | 1 | 1 | 1 | 1 |
| ENSG00000163817 | SLC6A20 | -0.210 | 0.310 | 1.000 | 1 | 0 | 1 | 0 |
| ENSG00000101350 | KIF3B | -0.209 | 0.343 | 1.000 | 2 | 1 | 1 | 0 |
| ENSG00000146376 | ARHGAP18 | -0.206 | 0.428 | 1.000 | 1 | 1 | 0 | 0 |
| ENSG00000151690 | MFSD6 | -0.203 | 0.335 | 1.000 | 1 | 1 | 1 | 1 |
| ENSG00000179295 | PTPN11 | -0.202 | 0.415 | 1.000 | 3 | 2 | 0 | 0 |
| ENSG00000153339 | TRAPPC8 | -0.197 | 0.391 | 1.000 | 1 | 1 | 1 | 1 |
| ENSG00000145782 | ATG12 | -0.197 | 0.352 | 1.000 | 1 | 0 | 1 | 0 |
| ENSG00000134684 | YARS | -0.197 | 0.342 | 1.000 | 2 | 0 | 1 | 0 |
| ENSG00000204866 | IGFL2 | -0.197 | 0.336 | 1.000 | 3 | 1 | 1 | 0 |
| ENSG00000171843 | MLLT3 | -0.194 | 0.363 | 1.000 | 1 | 1 | 0 | 0 |
| ENSG00000124422 | USP22 | -0.191 | 0.418 | 1.000 | 3 | 1 | 1 | 1 |
| ENSG00000072210 | ALDH3A2 | -0.190 | 0.398 | 1.000 | 2 | 1 | 1 | 0 |
| ENSG00000115275 | MOGS | -0.187 | 0.394 | 1.000 | 2 | 1 | 1 | 1 |
| ENSG00000124098 | C20orf108 | -0.184 | 0.382 | 1.000 | 1 | 0 | 1 | 0 |
| ENSG00000174796 | THAP6 | -0.184 | 0.414 | 1.000 | 2 | 0 | 1 | 0 |
| ENSG00000157593 | SLC35B2 | -0.184 | 0.389 | 1.000 | 1 | 1 | 0 | 0 |
| ENSG00000186260 | MKL2 | -0.181 | 0.373 | 1.000 | 3 | 0 | 2 | 0 |
| ENSG00000138593 | SECISBP2L | -0.181 | 0.395 | 1.000 | 1 | 1 | 1 | 1 |
| ENSG00000101146 | RAE1 | -0.178 | 0.414 | 1.000 | 1 | 1 | 0 | 0 |
| ENSG00000119314 | ROD1 | -0.175 | 0.437 | 1.000 | 1 | 1 | 1 | 1 |
| ENSG00000013588 | GPRC5A | -0.172 | 0.472 | 1.000 | 3 | 1 | 0 | 0 |
| ENSG00000015171 | ZMYND11 | -0.171 | 0.436 | 1.000 | 1 | 1 | 1 | 1 |
| ENSG00000095261 | PSMD5 | -0.168 | 0.431 | 1.000 | 2 | 1 | 1 | 0 |
| ENSG00000112308 | C6orf62 | -0.166 | 0.466 | 1.000 | 1 | 0 | 1 | 0 |
| ENSG00000177469 | PTRF | -0.165 | 0.440 | 1.000 | 3 | 0 | 1 | 0 |
| ENSG00000092140 | G2E3 | -0.161 | 0.515 | 1.000 | 1 | 0 | 1 | 0 |
| ENSG00000126878 | AIF1L | -0.160 | 0.453 | 1.000 | 2 | 2 | 0 | 0 |
| ENSG00000134352 | IL6ST | -0.159 | 0.500 | 1.000 | 2 | 1 | 0 | 0 |
| ENSG00000136875 | PRPF4 | -0.158 | 0.539 | 1.000 | 1 | 0 | 1 | 0 |
| ENSG00000065457 | ADAT1 | -0.157 | 0.469 | 1.000 | 2 | 0 | 1 | 0 |
| ENSG00000065548 | ZC3H15 | -0.157 | 0.459 | 1.000 | 1 | 0 | 1 | 0 |
| ENSG00000104738 | MCM4 | -0.153 | 0.531 | 1.000 | 1 | 0 | 1 | 0 |
| ENSG00000173141 | MRP63 | -0.153 | 0.508 | 1.000 | 4 | 0 | 2 | 0 |
| ENSG00000182795 | C1orf116 | -0.153 | 0.457 | 1.000 | 1 | 0 | 1 | 0 |
| ENSG00000156675 | RAB11FIP1 | -0.152 | 0.462 | 1.000 | 4 | 1 | 1 | 1 |
